# *Staphylococcus aureus* induces DNA damage in host cell

**DOI:** 10.1038/s41598-019-44213-3

**Published:** 2019-05-22

**Authors:** Martine Deplanche, Nassim Mouhali, Minh-Thu Nguyen, Chantal Cauty, Frédéric Ezan, Alan Diot, Lesly Raulin, Stephanie Dutertre, Sophie Langouet, Patrick Legembre, Frederic Taieb, Michael Otto, Frédéric Laurent, Friedrich Götz, Yves Le Loir, Nadia Berkova

**Affiliations:** 1 STLO, INRA, Agrocampus Ouest, Rennes, France; 2 Microbial Genetics, University of Tübingen, Tübingen, Germany; 3 Univ Rennes, Inserm, EHESP, Irset UMR_S 1085, F-35000 Rennes, France; 4 Centre International de Recherche en Infectiologie, INSERM U1111, CNRS, Université Lyon 1, Lyon, France; 5 Centre National de Référence des Staphylocoques, Lyon, France; 6 CNRS, Inserm, BIOSIT-UMS 3480, MRic, Université de Rennes, Rennes, France; 7 Centre Eugène Marquis, Equipe Ligue Contre Le Cancer, Rennes, France; 8 IRSD, Université de Toulouse, INSERM, INRA, ENVT, UPS, Toulouse, France; 9 Laboratory of Human Bacterial Pathogenesis, US National Institutes of Health, Bethesda, Maryland 20892 USA

**Keywords:** Cellular microbiology, Infection, Double-strand DNA breaks, Mechanisms of disease

## Abstract

*Staphylococcus aureus* causes serious medical problems in human and animals. Here we show that *S. aureus* can compromise host genomic integrity as indicated by bacteria-induced histone H2AX phosphorylation, a marker of DNA double strand breaks (DSBs), in human cervix cancer HeLa and osteoblast-like MG-63 cells. This DNA damage is mediated by alpha phenol-soluble modulins (PSMα_1–4_), while a specific class of lipoproteins (Lpls), encoded on a pathogenicity island in *S. aureus*, dampens the H2AX phosphorylation thus counteracting the DNA damage. This DNA damage is mediated by reactive oxygen species (ROS), which promotes oxidation of guanine forming 7,8-dihydro-8-oxoguanine (8-oxoG). DNA damage is followed by the induction of DNA repair that involves the ATM kinase-signaling pathway. An examination of *S. aureus* strains, isolated from the same patient during acute initial and recurrent bone and joint infections (BJI), showed that recurrent strains produce lower amounts of Lpls, induce stronger DNA-damage and prompt the G2/M transition delay to a greater extent that suggest an involvement of these mechanisms in adaptive processes of bacteria during chronicization. Our findings redefine our understanding of mechanisms of *S. aureus*-host interaction and suggest that the balance between the levels of PSMα and Lpls expression impacts the persistence of the infection.

## Introduction

Human cells are permanently exposed to environmental and endogenous factors that induce DNA damage, thus affecting genomic integrity and contributing to ageing^1^. Exogenous inducers include chemicals, radiations and various pathogens, whereas among endogenous inducers, the most relevant are Reactive Oxygen Species (ROS)^2,3^. The host cells counteract the consequences of lesions by DNA damage response (DDR) and checkpoint systems that repair DNA structure to maintain genomic integrity and cell survival or to trigger senescence or cell death when DNA is irredeemably damaged^4^. On their side, pathogens develop multiple strategies to promote infections^5,6^ by interfering with survival pathways^7^ and/or suppressing the immune response of the host thus facilitating the establishment of chronic infections and promoting host cell transformation^8^. Moreover, bacteria can directly and indirectly (via ROS) damage host DNA of which Double strand breaks (DSBs) are the most deleterious^9–11^. A deleterious action on host DNA integrity has been described for Gram-negative bacteria such as *Helicobacter sp*., *Chlamydia sp*., *Salmonella sp*., or *Escherichia coli*, demonstrating that these mechanisms may lead to genomic alterations and transformation associated with cancer development^5,10,12,13^. Rai *et al*. showed that Gram-positive bacterium *Streptococcus pneumoniae* induces a DSB and that Streptococcus pyruvate oxidase (SpxB) and a cholesterol-dependent cytolysin (CDC) toxin pneumolysin play a critical role in inducing DSBs^14,15^. However, such action has never been investigated for the Gram-positive bacterium, *S. aureus*. Yet, *S. aureus*-induced diseases represent serious clinical problems, especially during chronic infections^16^. In the case of chronic disease, *S. aureus* infections persist asymptomatically with relapses happening several months after optimal treatments even in immune-competent patients^17–20^. It implies that bacteria subvert the host cells defense functions for their own benefit^21,22^. Recent findings revealed that chronicization of *S. aureus* strains during bone and joint infections (BJI) leads to a phenotypical adaptation from a highly virulent to a less virulent form, which are frequently distinguished by an increased intracellular persistence and by their capacity to induce a lower level of cytokines release^23^. An example for such attenuated persisters are the so-called small colony variants (SCV)^20,24–26^.

The versatility of *S. aureus* arises from the multiplicity of virulence factors, which are extremely heterogeneous in structure and mode of action. Some virulence factors target the host cell membrane (e.g. pore forming toxins), tissue integrity (e.g. exfoliative toxins), or are involved in tissue colonization (e.g. adhesins)^27^. *S. aureus* can also target host cell activities such as cytoskeletal organization or cell cycle progression^28,29^. ROS that are generated by the host during infection^30^ can lead to the formation of deleterious oxidative host DNA lesions^31^ from which the most common one is 7,8-dihydro-8-oxoguanine (8-oxoG)^32,33^. Additionally to their molecular damage capacity ROS possess dramatically different opposed features such as regulators of signaling pathways^3^. While ROS induction by *S. aureus* was described in infected osteoblast-like SAOS-2 cells^34^, the *S. aureus*–induced DNA damage was not demonstrated and the underlying mechanism was not investigated.

Alteration of the host cell cycle by bacterial cyclomodulins is a common strategy to promote infection^12^. Recently we demonstrated that *S. aureus* virulence factors PSMsα and membrane-anchored Lpls induced a G2/M transition delay^29,35^. *S. aureus*-induced cell cycle alteration was associated with an increased bacterial internalization and their intracellular proliferation as well as with the decreased production of antibacterial peptides by host cells^29,36^. However, the causal relationship between the *S. aureus*-induced delay and the DNA damage that potentially provokes this delay was not examined. In the current study, we thus expanded these findings and investigated as to whether *S. aureus* induces DNA damage in host cells.

Latest advances in the understanding of mechanisms of chronic infections show that chronicization of *S. aureus* strains during BJI was associated to phenotypical adaptation of bacteria resulting in a decreased virulence and a diminished ability of immune system stimulation^23^. Nevertheless, the effect of initial vs recurrent isolates on the host molecular machinery, which may lead to genomic instability of host cells, was not explored.

In the present study, we demonstrate that *S. aureus* induces ROS-mediated 8-oxoG associated DNA damage followed by DNA repair and identified PSMα and Lpls as effectors of this phenomenon, however with opposing outcomes. We highlighted the fact that clinical isolates from the same patient with acute initial and recurrent BJI possess different capacities to compromise their host genomic integrity; recurrent isolates induce stronger DNA-damage and prompt the cell cycle transition delay to a greater extent. Our results demonstrate that *S. aureus* can directly compromise the genomic integrity of its host cells and strongly suggest this mechanism is involved in the adaptive processes of bacteria during chronic infection emphasizing the biological significance of our findings.

## Results

### A long-term *S. aureus* infected cell culture as a model of chronic infection

**Figure 1:**
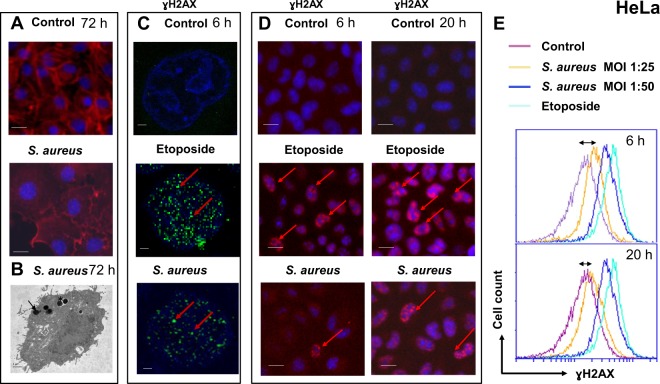
Exposure to *S. aureus* induces DNA damage in HeLa cells. (**A**) HeLa cells were infected with *S. aureus* MW2 strain at MOI 1:50 for 2 h. After fixation with 4% PFA, followed by permeabilization in 0.1%Triton/PBS solution cells were labeled with ActinRed™ reagent (TRITC-conjugated phalloidin that labels F-actin, red staining) and nuclei were labeled with DAPI (blue staining). Samples were viewed with a Zeiss fluorescence microscope using ×100 magnification. Overlaid fluorescent images of immunostained infected vs control non-infected HeLa cells (merged) are presented. Scale bar: 10 µm. (**B**) Transmission electron micrographs of HeLa cell infected with MW2 strain at MOI 1:50 for 72 h. Bacteria appear to be free within the cytoplasm (arrow) or in vacuoles (asterisk). Magnification x12,000, scale bar: 1 µm. (**C**) HeLa cells were infected for 6 h with MW2. Cells treated with 50 µM of etoposide, which induces DNA damage, were used as a positive control After fixation and permeabilization cells were stained for γH2AX, followed by incubation with Alexa Fluor 488 labeled secondary antibody (green staining). Nuclei were labeled with DAPI (blue staining). Samples were viewed with Leica SP8 laser-scanning microscope equipped with immersion objective 63× plan Apo-NA 1.4. Single nucleus (blue) with DNA containing γH2AX (green) in contrast to the negative control non infected cells are shown. Arrows show phosphorylated H2AX. Scale bar: 1 µm. (**D**) High Content Screening analysis of HeLa cells infected with MW2 for 6 h and 20 h. Immunolabelling of γH2AX with the ɣ-H2AX antibody followed by the incubation with the secondary antibody coupled with Alexa Fluor 555 (red staining) in HeLa cells exposed to *S. aureus* compared with that of the non-infected control cells. Nuclei were stained with DAPI (blue staining). Arrows show the site of phosphorylated H2AX. Fluorescence images were obtained with a Cellomics ArrayScan VTI HCS Reader. Scale bar: 10 µm. (**E**) HeLa cells were infected with *S. aureus* with MOI 1:25 and 1:50 for 4 h and 20 h. γH2AX was quantified by flow cytometry. The data were collected from 20,000 cells and analysis was performed with Cell Quest software. Double arrow shows the shift between the non-infected control cells (violet line) and cells infected with *S. aureus* at MOI 1:50 (yellow line).

Exposing HeLa cells to *S. aureus* MW2 (USA400) resulted in internalization of bacteria and in the enlargement of host cells (Fig. 1A), associated with a G2/M transition delay as shown previously^29,36^. In the present study, infected cells were observed by electron microscopy up to 15 days post-infection (Fig. 1B). Intracellular bacteria were found free within the cytoplasm (arrow) or entrapped in vacuoles (asterisk) (Fig. 1). Control non-infected cells showed longitudinal distribution of actin filaments, whereas *S. aureus*-infected cells showed changes of actin distribution (Fig. 1A), suggesting the involvement of actin remodeling in *S. aureus* infection.

### *S. aureus* induces ROS-mediated DNA damage and repair response in eukaryotic cells

We compared the phosphorylation of H2AX (ɣH2AX), which is a marker for DNA damage in the absence of apoptosis, in *S. aureus* MW2 infected *vs* non-infected cells. Immunofluorescence revealed that 6 h post-infection HeLa cells exhibited ɣH2AX in their nuclei, which extended up to 20 h post-infection (Fig. 1C,D). Apoptosis was not observed in these conditions since neither processed caspase-3, nor cleaved PARP, nor apoptotic bodies were detected in infected cells, while the active caspase-3, cleaved PARP and apoptotic bodies were observed in staurosporine-treated cells (Supplementary Fig. S1), suggesting that *S aureus*-induced DSBs result from genotoxic activity and not from apoptosis (or cell death).

These findings were confirmed by flow cytometry analysis. Six hours post-infection, the ɣH2AX signal was higher in infected cells than in uninfected ones (Fig. 1E). Twenty hours post-infection at MOI 1:25 cells showed a background level of ɣH2AX (Fig. 1E) suggesting, that infected cells withstand DNA damage by DNA repair mechanisms.

To assess whether *S. aureus* induces DNA damage in other cell types, we monitored ɣH2AX in osteoblast-like MG-63 cells. Similarly to HeLa cells S. aureus induces dose-dependent H2AX phosphorylation in MG-63 cells (Supplementary Fig. S2, Supplementary Table S3).

**Figure 2:**
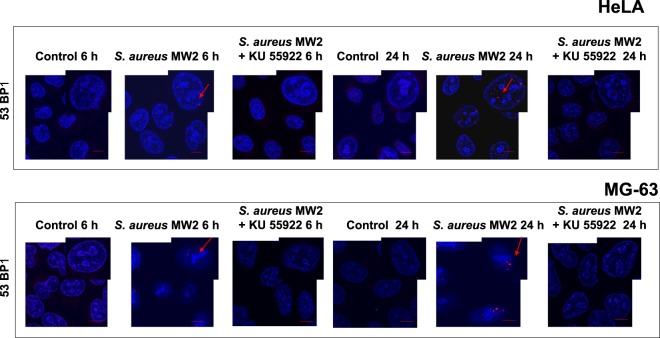
*S. aureus* infection induces DNA damage-response in HeLa and MG-63 cells. HeLa or MG-63 cells were infected with *S. aureus* MW2 strain (MOI 1:50) for 2 h as described. In some experiments, cells were treated with the ATM inhibitor KU-55933 during infection. After 6 h and 20 h post-infection, cells were immunostained for the Binding Protein, 53BP1, followed by incubation with anti-mouse IgG TRITC-labeled antibody (red staining, the red arrow). Nuclear DNA were labeled with DAPI (blue staining). Samples were viewed with Leica SP8 laser-scanning microscope equipped with immersion objective 63× plan Apo-NA 1.4driven by the LAS software. Scale bar: 5 µm.

To verify whether *S. aureus*-induced DSBs triggered DNA repair, we next monitored the nuclear staining of the early repair protein, 53BP1^10,37^. As shown by confocal microscopy the recruitment of 53BP1 to nuclear foci was observed 6 h post-infection and was further increased 24 h post infection (Fig. 2). To define whether the formation of 53BP1 foci was associated with a canonical DDR comprising the triggering of the ATM kinase-signaling pathway, HeLa and MG-63 cells were treated with the ATM inhibitor KU-55933. An addition of KU-55933 resulted in a strong decrease of the proportion of positive 53BP1-stained infected cells (Fig. 2).

**Figure 3:**
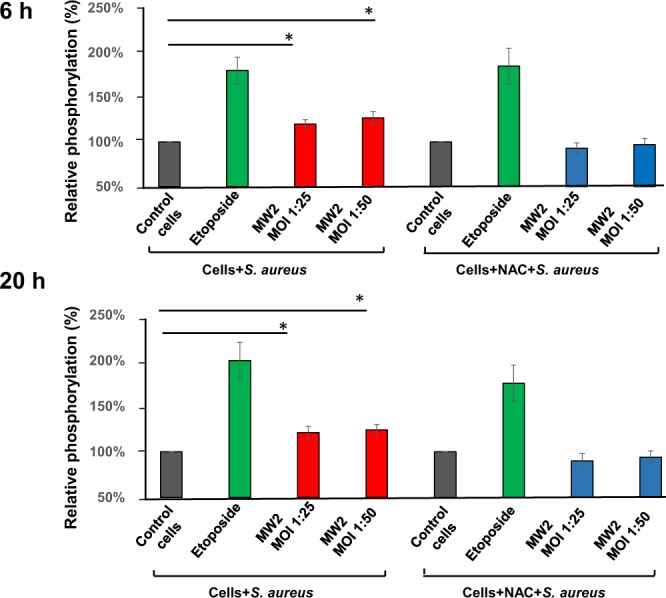
*S. aureus* induces a ROS-mediated host DNA damage. HeLa cells were exposed to *S. aureus* MW2 strain (MOI 1:25 and 1:50) for 2 h as described. In parallel, some Hela cells were treated with the ROS inhibitor NAC (N-acetyl-L-cysteine) 1 h before the exposure of cells to bacteria. As the positive control 50 µM etoposide was used. Either 6 h or 20 h post-infection phosphorylated H2AX was quantified by flow cytometry. The data were collected from 20,000 cells and analysis was performed with Cell Quest software. The relative phosphorylation of the control cells was considered as 100%. Percent of the relative phosphorylation of samples was calculated as fold changes over the control and multiplied by 100. Data are presented as mean ± SD from three independent experiments.

**Figure 4:**
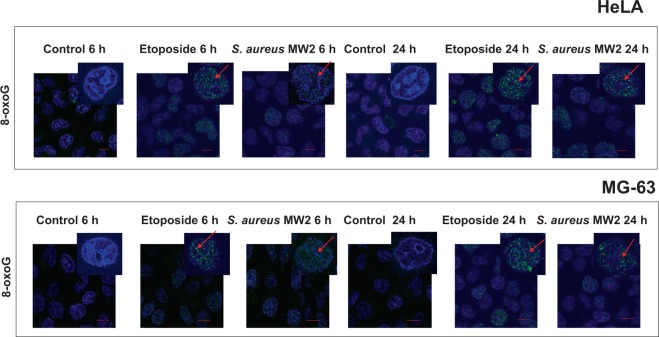
*S. aureus* induce 8-oxoG DNA lesion in the host cells. Either HeLa or MG-63 cells were exposed to *S. aureus* MW2 strain at MOI 1:50 for 2 h followed by antibiotic treatment as described. As the positive control 50 µM etoposide was used. After 6 h and 20 h of post-infection cells were immunostained with mouse anti 8-oxoG DNA lesion antibody, followed by incubation with m-IgGκ BP-FITC at dilution 1:50 for 2 h at room temperature (green staining, the red arrow). Nuclear DNA were labeled with DAPI (blue staining). Samples were viewed with Leica SP8 laser-scanning microscope equipped with immersion objective 63× plan Apo-NA 1.4 driven by the LAS software. Scale bar: 5 µm.

It is known that, in response to *S. aureus* infection, host cells synthesize ROS with potent cytotoxic properties against bacteria^34,38,39^. Since ROS can act as a second messenger in the regulation of signaling pathways as well as to trigger DNA damage in the host cells^3,5^, we investigated the possible implication of ROS in *S. aureus-*induced DNA damage by using the ROS inhibitor N-acetyl-L-cysteine (NAC). Incubation of host cells with NAC 1 h before exposure to *S. aureus* for 6 h and 20 h prevented the induction of the DNA damage (Fig. 3), showing that ROS is involved in *S. aureus*-induced DNA damage. To better understand the mechanism of *S. aureus* induced DNA damage, the mutagenic lesion 8-oxoG, which is most often involved in oxidative DNA damage, was monitored in infected HeLa and MG-63 cells. Both infected cells lines showed increased levels of 8-oxoG, indicating that *S. aureus* prompts a ROS production, which induces 8-oxoG DNA lesion (Fig. 4).

Altogether, these results indicated that *S. aureus* induces a production of ROS causing DNA damage that is followed by the canonical DDR in infected cells.

### Pivotal role of *S. aureus* PSMα_1–4_ toxins in the induction of host DNA damage

*S. aureus* PSMs have been shown by us to induce cell cycle arrest^29^. Because DNA damage induces cell cycle arrest to allow DNA repair, we examined whether PSMs are involved in *S. aureus*-mediated DNA damage.

**Figure 5:**
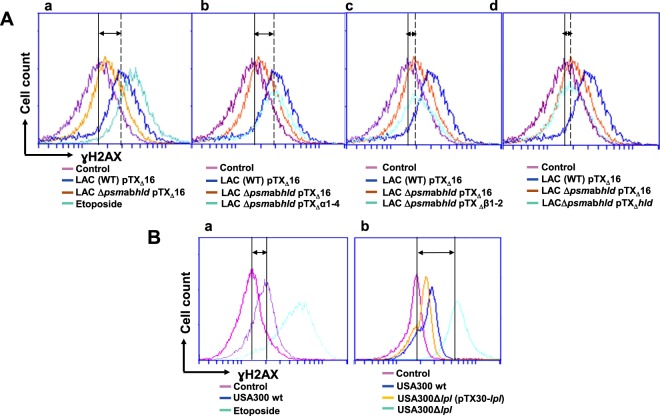
*S. aureus* PSMα_1–4_ toxins induce and Lpls dampen DNA damage of infected cells. (**A**) HeLa cells were infected either with *S. aureus* LAC (WT) pTX_∆_16 containing plasmid pTX_∆_16 (blue line), or with the PSM-deficient deletion mutant LAC Δ*psm*αβ*hld* pTX_∆_16 (yellow line), or with complemented mutant LACΔ*psm*αβ*hld* pTX_Δ_α1-4 (blue-green line, b), LACΔ*psm*αβ*hld* pTX_Δ_β (blue-green line, c) and LACΔ*psm*αβ*hld* pTX_Δ_hld (blue-green line d) at MOI 1:50 for 2 h as described. As a positive control 50 µM etoposide was used (blue-green line, a). At 20 h post infection phosphorylated γH2AX was quantified by flow cytometry. The data were collected from 20,000 cells and analysis was performed with Cell Quest software. Double arrow shows the shift between the control cells (violet line) and a corresponding mutant (b–d, blue-green line). One representative result is shown. (**B**) HeLa cells were infected either with USA300 wt (a, b, blue), or with the deletion mutant USA300Δlpl (b, blue-green) or with the complemented mutant USA300Δlpl (pTX30-*lpl*) (b, yellow) at MOI 1:50 for 2 h as described. At 20 h post infection phosphorylated γH2AX was quantified by flow cytometry. The data were collected from 20,000 cells and analysis was performed with Cell Quest software. Double arrow shows the shift between the control cells and USA300 (a) and between the control cells and USA300Δlpl (b). One representative result is shown.

We compared the capacity of the wild type *S. aureus* LAC (pTX_∆_16) containing plasmid pTX_∆_16 with those of the psm-deletion mutant, LAC Δ*psm*αβ*hld* (pTX_∆_16) to induce ɣH2AX phosphorylation by a flow cytofluorometry (Fig. 5Aa). The exposure of HeLa cells to LAC (pTX_∆_16) resulted in an increase in ɣH2AX phosphorylation: the peak of fluorescence was shifted to the positive control value of etoposide-treated cells. Cells exposed to the psm-deletion mutant, LACΔ*psm*αβ*hld* (pTX_∆_16), did not induce ɣH2AX phosphorylation: the peak of fluorescence was in the region of the control cells (Fig. 5Aa). To identify the PSM responsible for this DNA damage, histone ɣH2AX phosphorylation in the control cells was compared to that in cells exposed to the complemented mutants. There were no differences between the fluorescence of cells exposed to the mutant LAC Δ*psm*αβ*hld* (pTX_∆_16) and to the complemented mutants either LACΔ*psm*αβ*hld* (pTX_Δ_β) or LACΔ*psm*αβ*hld* (pTX_Δ_hld) (Fig. 5Ac,d). In contrast, the complemented mutant, LACΔ*psm*αβ*hld* (pTX_Δ_α1-4), induced a shift of fluorescence which was comparable to that induced by LAC (pTX_∆_16) (Fig. 5Ab). Altogether, these results suggest the pivotal role of PSM_α1–4_ in *S. aureus*-induced DNA damage.

### Lpl lipoproteins dampen the *S. aureus*–induced DNA damage

We recently demonstrated that Lpls cause host cell cycle arrest^35^. To investigate the effect of Lpls on the integrity of the host DNA we compared the H2AX phosphorylation in cells exposed to USA300 wild type with those of the deletion strain USA300Δ*lpl* or the complemented USA300Δ*lpl* (pTX30-*lpl*) mutants using flow cytometry (Fig. 5Ba). The exposure of HeLa cells to USA300 wt engendered an increase in H2AX phosphorylation as demonstrated by the shift of the peak of fluorescence towards the value of etoposide-treated cells (Fig. 5B). The peak in cells exposed to the deletion USA300Δ*lpl* mutant was shifted to an even higher extent. Exposure to the complemented USA300Δ*lpl* (pTX30-*lpl*) mutant decreased H2AX phosphorylation: the peak of fluorescence remained closer to the level of the USA300 wt (Fig. 5Bb). According to three independent experiments, exposure of HeLa cells to USA300 wt (MOI 1:50) resulted in an increase of normalized fluorescence related to H2AX phosphorylation from 100% (control) to 110 ± 4% (Supplementary Fig. S4). Exposure of HeLa cells to the deletion mutant USA300Δ*lpl* (MOI 1:50) increased normalized fluorescence up to 127 ± 5%, while exposure to the complemented mutant USA300Δ*lpl* (pTX30-*lpl*) (MOI 1:50) decreased the normalized fluorescence to 111 ± 4%, at the level of the USA300 wt (Supplementary Fig. S4). These results indicate that Lpls dampen the DNA damage.

### Recurrent *S. aureus* isolates induce a stronger DNA damage and produce lower amount of Lpls than initial clones

So far we have investigated the effect of *S. aureus* on the host cell DNA damage with clinical isolates that have been extensively passaged in laboratories. To better understand whether the induction of DNA damage is a common feature of *S. aureus* or is limited to certain strains, and evaluate the biological significance of the described phenomenon, we tested the effect with freshly isolated clinical strains, which were obtained from patients that were diagnosed with initial acute vs recurrent BJI^23^. Isolates of the same patient were sampled at initial episode of infection and after delayed recurrent BJI. It has been demonstrated that recurrent isolates induce a reduced inflammatory response and less mortality in a mouse model when compared to initial isolates^23^. In the present study DNA damage of host cells was estimated by analyzing phosphorylated ɣH2AX using flow cytometry and immunofluorescence method. According to the flow cytometry analysis, all strains significantly induced DNA damage, however, to a different extent. Initial isolates 45i (P1), 47i (P2) and 51i (P3) induced less DNA damage than the recurrent 46r (P1), 48r (P2) and 52r (P3) isolates (Table 1).

**Table 1:** *S. aureus* isolates from patients with a relapsing BJI induce a stronger DNA damage than isolates from patients with an initial BJI.

Conditions	Relative phosphorylation 6 h		Relative phosphorylation 20 h	
Samples	MOI 1 :25	MOI 1 :50	MOI 1 :25	MOI 1 :50
Control non-infected HeLa cells	100%		100%	
Hela cells + Patient 1 Initial infection strain 45i	107 ± 4%*	111 ± 7%*	106 ± 5%*	117 ± 5%*
Cells + Patient 1 Recurrent infection strain 46r	117 ± 6%*	125 ± 6%*	120 ± 6%*	132 ± 8%*
Control non-infected HeLa cells	100%		100%	
Cells + Patient 2 Initial infection strain 47i	108 ± 4%	113 ± 7%	109 ± 4%*	110 ± 6%*
Cells + Patient 2 Recurrent infection strain 48r	114 ± 6%	117 ± 10%	117 ± 6%*	125 ± 5%*
Control non-infected HeLa cells	100%		100%	
Cells + Patient 3 Initial infection strain 51i	106 ± 4%*	113 ± 7%*	107 ± 4%*	114 ± 7%*
Cells + Patient 3 Recurrent infection strain 52r	118 ± 6%*	130 ± 7%*	119 ± 6%*	139 ± 8%*

Relative phosphorylation in HeLa cells was obtained using flow cytometry analysis. Normalization was performed as follows: the percent of relative phosphorylation of clinical isolates was calculated as fold changes over the one of control cells that was considered as 100% and multiplied by 100. P ≤ 0.05 was considered significant. The differences among the groups were assessed by ANOVA. Tukey’s honestly significant difference test was applied for comparison of means between groups. The values are expressed as mean ± SD.

*P ≤ 0.05 relative phosphorylation induced by strains from recurrent BJI vs. relative phosphorylation induced by strain isolated from initial BJI (the same patient).

**Figure 6:**
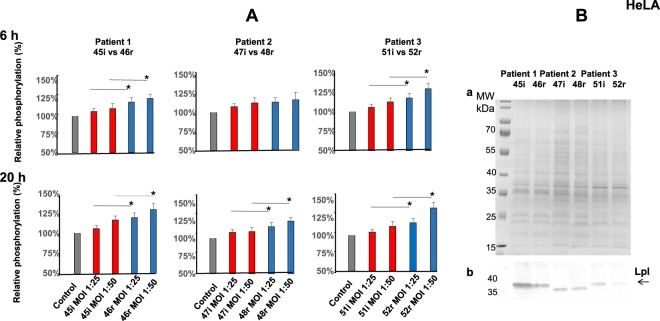
*S. aureus* recurrent isolates induce stronger DNA damage in HeLa cells and express a lower amount of Lpls than initial acute isolates. (**A**) HeLa cells were exposed for 2 h to tree couples of isolates (MOI 1:25 and 50) that have been recovered from three patients (P1, P2, P3) at the time of the initial (45i, 47i, 51i, red) and relapsing (46r, 48r, 52r, blue) infection from P1, P2, P3 correspondently. At 6 h and 20 h post-infection phosphorylated γH2AX was quantified by immunofluorescence analysis using High Content Screening approach as described in Material and Methods. DNA and ɣH2AX-immunofluorescence were visualized using a Cellomics ArrayScan VTI HCS Reader (Thermo Fisher) ImPACcell technologic platform. Ten high-definition images per well of a 96 multiwall plate were analyzed and an arbitrary immunofluorescence value was found for each nucleus. Results are expressed as a percentage of nucleus labelled with ɣH2AX. The relative phosphorylation of the control cells was considered as 100%. Percent of the relative phosphorylation of samples was calculated as fold changes over the control and multiplied by 100. Data are presented as mean ± SD from three independent experiments. P-values, 0.05 (*) were considered to be significant. (**B**) *S. aureus* strains isolated from three patients (P1, P2, P3) with initial (45i, 47i, 51i) and recurrent (46r, 48r, 52r) BJI from P1, P2, P3 correspondently were maintained as described. Lpls enriched fractions were prepared as indicated in Material and Methods. TritonX114 lipoprotein-enriched fractions, prepared from 6 clinical isolates, were separated on 12% SDS-PAGE: (a) Gel was stained with Coomassie blue (b) The Western blot analysis was performed using anti-Lpl1-his antibody which we developed. Molecular Weight markers are presented at the left side of SDS-PAGE gel and membrane. The arrow indicates *S. aureus* LpLs.

These observations were confirmed through the quantification of positive-stained MG-63 cells by High Content Screening analysis (Fig. 6A, Supplementary Fig. 5).

The comparison of the H2AX phosphorylation revealed that recurrent *S. aureus* isolates induced greater DNA damage than initial acute isolates (Fig. 6A, Table 1). At 6 h post-infection, there was a significant difference in the H2AX phosphorylation induced by 45i vs 46r and by51i vs52r strains. At 20 h post-infection there was a significant difference in the H2AX phosphorylation induced by recurrent vs initial isolates obtained from all three patients. Similar results were obtained with MG-63 (Supplementary Fig. 5).

As on the one hand *S. aureus* Lpls dampened bacteria-induced DNA damage and on the other hand the capacity to induce DNA damage was different in the strains isolated from patients with an initial and recurrent BJI, we estimated Lpls expression in these isolates. The *lpl* cluster contained different numbers of *lpl* genes in various *S. aureus* strains. However, Lpl protein sequences share >60% similarity, and the core region even 90% similarity^40,41^. Therefore, polyclonal anti-Lpl1-his antibodies, cross-react with the various Lpl proteins^40,42^. TritonX114 lipoprotein-enriched fractions were prepared from three couples of clinical isolates, which were sampled from patients with an initial acute or relapsing BJI, and were grown under identical conditions as described in Materials and Methods. The same amount of TritonX114 lipoprotein-enrich fragments of each pairs were subjected to SDS-PAGE and subsequently Coomassie Blue stained (Fig. 6Ba). The protein patterns and the protein loads were similar within each couple of isolates. However, Western blot analysis with anti-Lpl1-antibodies revealed a clear difference in expression of Lpls between the pair 45i and 46r and the pair 51i and 52r and faint one between 47i and 48r (Fig. 6Bb, Supplementary Fig. S6). This suggests that the Lpl production was stronger in initial compared to recurrent isolates. Collectively these results suggested that recurrent *S. aureus* isolates induced a stronger DNA damage than initial ones and this capacity could be linked at least partially to the low level of Lpls production.

### Recurrent *S. aureus* isolates induce a stronger G2/M phase delay than initial acute isolates

**Figure 7:**
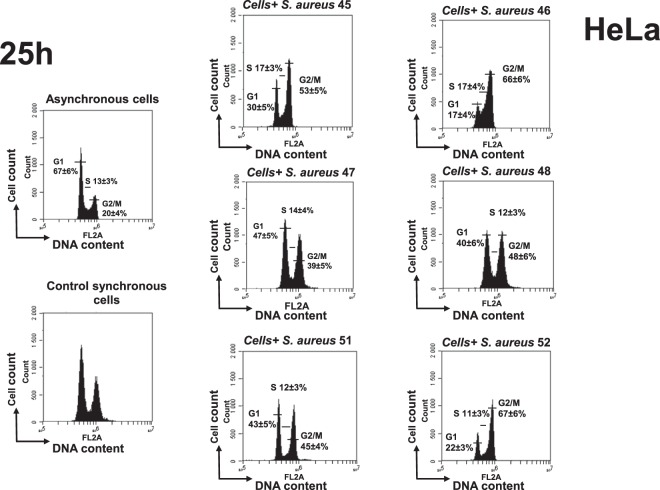
*S. aureus* recurrent isolates induce a stronger G2/M phase transition delay than initial acute isolates. HeLa cells were synchronized by DTB and were exposed for 2 h to *S. aureus* strains (MOI 1:50) isolated from three patients (P1, P2, P3) with initial acute (45i, 47i, 51i) and recurrent (46r, 48r, 52r) BJI from P1, P2, P3 correspondently. Cell cycle phase distribution was determined 25 h post infection by flow cytometry. The data were collected from 20,000 cells and analysis was performed with Cell Quest software. Each experiment was performed three times. The number of cells in different phases is presented on the histograms. The data correspond to a representative experiment out of the three assays performed and are presented as mean ± SD.

Since, on the one hand, recurrent and initial *S. aureus* isolates induced DNA damage to a different extent and, on the other hand, DNA damage could lead to the arrest of cell cycle progression^43^, we next evaluated the alteration of host cell cycle progression induced by those strains. At 25 h post-infection the distribution of HeLa cell cycle phases of control cells was 38 ± 6.1% (G1 phase), 34 ± 3.9% (S phase) and 28 ± 4.3% (G2/M phase) indicating that cells completed the first cell cycle and progressed within the second (Supplementary Table S7). The comparison of the effect of recurrent and initial isolates indicated that recurrent isolates slowed down host cell cycle progression to a greater extent than the initial strains (Fig. 7, Supplementary Table S7).

## Discussion

The damage of nuclear DNA in eukaryotic cells induced by bacteria is poorly documented and most studies belong to the field related to gastro-intestinal tract pathogens^5,12^.

Chronic *S. aureus* infection is likely to be associated with the internalization of the pathogen by host cells, where bacteria are protected from host defenses and therapy^44^. Consequently, bacteria can persist inside host cells for months or even years and induce only a low-grade inflammation and limited clinical signs^8,21^. The relapse can occur many months after the initial episode, even in immunocompetent patients^19^. In this work, using the model of long-term intracellular *S. aureus* infection we detected bacteria within the cytoplasm and in vacuoles in agreement with the previous report^45^. In contrast to the control, *S. aureus*-infected cells lost longitudinal distribution of actin filaments in agreement with data showing that the proper assembly of F-actin and microtubule network were essential for *S. aureus* internalization^46,47^. Actin rearrangements are also observed in *Mycobacterium*-infected cells^48^ suggesting the universality of the involvement of actin in host cell response to different internalized pathogens.

To evaluate the integrity of DNA in infected cells, we assessed ɣH2AX phosphorylation, a marker for DSBs-associated DNA damage in the absence of apoptosis^49^. The capacity to induce dose-dependent DSBs in epithelial and osteoblastic cell lines was shared by various virulent *S. aureus* strains including methicillin resistant MW2 (USA400), USA300 strains as well as clinical isolates obtained from patients with initial acute and recurrent BJI. However, the phosphorylation was increased to a different extent suggesting a strain-dependent capacity to induce DNA damage. The damaged DNA triggers a DDR involving the recruitment of the early repair factor 53BP1 that promotes the end-joining of distal DNA ends^37^. *S. aureus*-infected cells showed a nuclear staining of 53BP1 at 6 h post-infection that increased over time. It suggests that the formation of 53BP1 foci is the result of a physiological response to DSBs, which is mediated by the activation of the ATM kinase at damaged DNA sites^50^.

Following pathogen infection, the host defense system induces the production of small reactive molecules, such as ROS, that eradicate pathogens, but also trigger DNA damage in the host cells^5,51^. It was shown that internalized *S. aureus* induces ROS in human osteoblast-like SAOS-2 cells^34^. However, ROS can be triggered by the infection without contributing to the DSB formation, as was reported for *H. pylori*^10^. In this work using a ROS inhibitor, we established that ROS are critical for ɣH2AX phosphorylation, indicating that *S. aureus* induced ROS-dependent DNA damage. Among multiple ROS-induced DNA modifications, 8-oxoG DNA lesions are the most common lesions. These lesions can generate DNA double strand breaks when they occur during DNA replication and are thus exceptionally deleterious^33^. Results of the current study using two different cell lines suggest that *S. aureus* stimulates ROS production followed by the induction of 8-oxoG resulting in DSB of host cells.

DNA damage of eukaryotic cells may reversibly arrest cell cycle progression to allow DNA repair^52,53^. In addition to DNA damage, cell cycle arrest may be associated with the actin state organization^54,55^. Cyclomodulins can prompt a genotoxic effect by inducing DSBs or by activating the DNA damage checkpoint pathway that leads to the cell cycle arrest as was revealed for *E. coli* polyketide megasynthases^12^. Previously we have demonstrated that *S. aureus* virulence factors PSMs and Lpls possessed properties of cyclomodulins since they induce the G2/M transition delay in infected cells^29,35^. In the current study we examined the capacity of PSMs and Lpls to trigger DNA damage and we identified PSM_α1–4_ as essential contributors to this phenomenon. Our results are in line with previous observations by Forsman *et al*.^56^, which showed that the recognition of PSMα peptides by the formyl peptide receptor 2 (FPRP2) promotes neutrophils to produce ROS which in turn trigger inactivation of PSMs peptides through oxidation. Furthermore, independently of FPRP2 PSMs transferred cells to a viable, but dysfunctional state characterized by the diminished response to other ROS-releasing stimuli. Other researchers have also shown that the ligands of the intracellular receptor TLR9 like the synthetic CpG-motifs containing oligodeoxynucleotide (CpG-ODNs), promote killing of *S. aureus* inside the SAOS-2 osteoblast-like cell line by inducing ROS production^34^. These two studies clearly show that PSMα peptides and CpG-nucleotides contribute to ROS formation. Based on our and other researchers’ results^56^, we suggest that PSMs induce ROS that lead to 8-oxoG-associated host DNA damage.

Our previous investigations revealed causal consequences of *S. aureus*-induced G2/M delay: the G2 phase is advantageous for bacterial intracellular replication and is associated with a decreased production of antibacterial peptides that may contribute to the persistence of the infection^29,36^. Here we obtained a more complete picture of subversion of host cell functions by *S. aureus* during persistence.

Other important virulence factors are the Lpls (*lpl* gene cluster is localized within the νSaα pathogenic island) that express cyclomodulin activities^35^ and that are responsible for an increased invasion and an increased bacterial burden in a mice infection model^40^. Here, Lpls have been found to dampen DNA damage. The dissimilar effect of PSM_α1–4_ and Lpls on DNA-damage, despite the common capacity to induce the G2/M phase delay, may be due to the different mechanism of the delay and to their divergent properties. It has been demonstrated in this study that DNA damage of eukaryotic cells may transiently arrest the cell cycle progression to allow DNA repair^52,53^. Cell cycle arrest also may be associated with the actin state organization^54,55^. Previously we have shown that the G2/M phase transition is significantly delayed by the USA300 wild type, while the deletion mutant USA300∆*lpl* induces a lower transition delay^35^. We assume that PSM_α1–4_ induce DNA damage, while Lpls induce an actin rearrangement both resulting in the G2/M phase delay. In both cases, pathogen invasion is boosted and leads to an increased bacterial burden.

Pathogen-induced DSBs followed by an inaccurate DNA repair are generally associated with carcinogenic and mutagenic properties *in vitro* and *in vivo* of many bacteria such as *H. pylori*, *E. coli* or others^12,57^. Thereafter, *S. aureus*-induced DNA-damage may induce an environment in the host that is favorable to the development of different kind of pathologies including malignancies. Recent investigations show the connection between transcription and DNA breaks: the authors proposed a novel mechanism of transcriptional activation, which requires H2AX phosphorylated at S139 (ɣH2AX)^58,59^. Hence, we assume that in addition to its capacity of compromising genomic integrity of host cells, *S. aureus*-induced DNA damage could regulate a gene transcriptional activation in infected host cells through H2AX phosphorylation.

The biological significance of the current findings was demonstrated using freshly collected *S. aureus* clinical isolates. We recently described adaptive processes of *S. aureus* isolates during the progression from acute to chronic BJI by comparing initial and recurrent isolates from the same patient^23^. In the present study, using the same clinical isolates we found that recurrent isolates induce more DNA breaks and a higher G2/M phase delay compared to initial isolates.

Remarkably the recurrent isolates expressing lower amounts of Lpls cause a stronger DNA damage and a stronger G2/M phage delay than the initial acute isolates. We assume that the recurrent isolations contain factors other than PSMs and Lpls, which interfere with the cell cycle progression. Indeed, we identified that the initial and recurrent isolates differ by very few SNPs at the genomic level^23^. It would thus be interesting to know if these SNPs are located in regulatory regions of protein-coding RNAs or non-coding RNAs.

Our results suggest that *S. aureus* triggers ROS-mediated DNA damage thus affecting the genomic integrity and/or regulating a gene transcriptional activation, that induced DNA damage depends on the balance between the levels of the expression of PSMα and Lpls and that bacterial adaptation during chronicization is linked to the maintenance of the host genomic integrity. It would be interesting to investigate such phenomenon *ex vivo*, using primary cell culture, and *in vivo* using either biological samples from patients who have an *S. aureus* infection or from *S. aureus*-infected animal models.

These findings open a new avenue for the development of the innovative therapeutic strategies that either suppress DNA damage or boost DNA repair during *S. aureus* infection.

## Materials and Methods

### Maintenance of eukaryotic cells

The human cervix cancer HeLa (ATCC) and osteoblast-like MG-63 (LGC Standards, Teddington, UK) were used in this study. The objective of the present study is to investigate the effect of *S. aureus* infection on non-professional phagocytes. HeLa cells are widely used to investigate cell cycle arrest and DNA damage and repair^60^. Cancerous MG-63 cells are usually used as a model to study osteoblast infection^61^. Cells were cultured in cDMEM (DMEM, GlutaMax, 10% fetal calf serum (Gibco) supplemented with 100 U/ml penicillin, and 100 µg/ml streptomycin at 37 °C; trypsin/EDTA (Sigma) was used for cells subculturing.

### *S. aureus* strains description

Clinical isolates were collected with the approval of the French South-East ethics committee (no. CAL2011-21)^23^. Three couples of isolates were selected from patients P1, P2, P3 who were diagnosed with initial acute (i) and recurrent (r) staphylococcal BJI: isolates from the same patient were named 45i and 46r (P1), 47i and 48r (P2), 51i and 53r (P3) for initial and recurrent BJI correspondently. The strains were analyzed previously^23^. Briefly, 46r differed from 45i isolate by SNP mutations located in 4 intergenic regions, non-synonymous SNP in the intragenic region of 3 hypothetical proteins and 1 synonymous SNP in the DeoC encoding gene, which does not affect the primary amino acid sequence. 48r differed from 47i by SNP in 6 intergenic regions, 1 non-synonymous SNP in the intragenic region of an unknown protein, and 1 indel leading to a frameshift in the comK gene. 52r differed from 51i by SNP in 3 intergenic regions, and indels in 3 intragenic regions (encoding EssC, GTP-binding protein TypA/BipA-like protein, and a uridine kinase). Beside these differences, the genomic comparison did not reveal mutations in the major regulatory systems (*agr*, *sar*, *sigma* genes) or in virulence genes. The recurrent isolates persisted longer in the osteoblasts, expressed the lower cytokines level and were less virulent in mouse model, compared to the initial isolates.

The methicillin-resistant strains *S. aureus* MW2 (USA400), USA300 wild type and USA300 LAC (pTX∆16) which carries the control plasmid, the deletion mutant LAC∆psmαβhld (pTX∆16) and the complemented strains expressing either the four PSMα peptides (LAC∆*psm*αβ*hld*-pTX_∆_α1-4), PSMβ (LAC∆*psm*αβ*hld*-pTX_∆_β1-2), or *hld* (LAC∆*psm*αβ*hld*-pTX_∆_*hld*) were obtained from the Laboratory of Bacteriology, NIH, USA^62^. The pTX_Δ_ plasmids were derived from plasmid pTX15 with the deletion of the *xylR* repressor gene. Construction of the mutant USA300∆*lpl* in which the entire *lpl* cluster was deleted and the complemented mutant USA300∆*lp*l (pTX30-*lpl*) was described^40^. The tetracycline-resistant strains harboring plasmid pTX∆16 were grown in BHI containing 12.5 µg/ml of tetracycline.

Aliquots from overnight cultures on Brain Heart Infusion (BHI) broth were diluted (1:50) in DMEM. The growth curves of mutants were similar to that of the wild type. Strains were grown at 37 °C under anaerobic conditions until cultures reached an optical density of 0.6 at 600 nm, corresponding to 10^8^ CFU/ml. Bacterial concentrations of staphylococci in the interaction medium (DMEM) were estimated spectrophotometrically and were confirmed by plate counts.

### Cell synchronization

The double thymidine block (DTB) was used to synchronize cells^29,35^. Briefly, cells were grown up to 30% confluence^63^ and were cultivated in cDMEM containing 2 mM thymidine (cDMEM-T) for 18 h, followed by cultivation in cDMEM for 9 h. Afterwards, cells were cultivated in cDMEM-T for 17 h, followed by cultivation in cDMEM. Duration of HeLa cell cycle phases is 10, 8, 3, and 1 h, for G1, S, G2, and M, respectively^64^.

### Cell culture infection

For the cell cycle analysis, synchronized HeLa cells at the border of G1/S phase were infected with *S. aureus* with a multiplicity of infections (MOI, number of bacteria per cell) 50:1, 3 h after DTB release^29,35,36^. Extracellular bacteria were removed 2 h post-infection by incubation cells in cDMEM with 20 μg/ml of lysostaphin and 100 μg/ml gentamicin for 2 h, followed by incubation in cDMEM containing 25 μg/ml of gentamicin. For the analysis of ɣ-H2AX asynchronous cells were used.

### Transmission electron microscopy

HeLa cells were fixed in 2.5% glutaraldehyde/cacodylate buffer; then in 1% osmium tetroxide. The pellet was mixed with 3% agar in sodium cacodylate, 7,3 dehydrated, embedded in Epon-Araldite-DMP30 resin mixture and polymerized at 60 °C for 48 h. Sections were cut in Leica ultra microtome, stained with uranyl acetate and were analyzed with JEOL 1400 Electron Microscope (Jeol, Tokyo, Japan). Images were digitally captured with GATAN Orius camera (Digital Micrograph Software).

### Confocal and fluorescence microscopy

Cells were grown on cover slips then were exposed to MW2 for 2 h. 25 h post-infection cells were fixed with 4% paraformaldehyde/PBS for 20 min, followed by permeabilisation in 0.1%Triton/PBS and incubation with 20% goat serum (Sigma). Rabbit anti-human phospho-histone H2AX (Ser 139) (Cell Signaling, USA) or mouse anti-human 53BP1 (E-10) (Santa Cruz, USA) 1:100 in 1% BSA/PBS were applied overnight at 4 °C, followed by incubation either with Alexa Fluor 488 labeled goat anti-rabbit antibody (Cell Signaling) (1:1000) or anti-mouse TRITC-labeled antibody (Sigma) (1:100) for 2 h. In some experiments, cells were pretreated with 10 μM of ATM inhibitor KU-55933 (Calbiochem) before infection. For 8-oxoG immunostaining cells were incubated with mouse anti-8-oxo DNA lesion antibody (483.15: sc-130914, Santa Cruz, USA),(1:100), followed by incubation with mouse IgGκ light chain binding protein conjugated to fluorescein isothiocyanate (sc-516140, Santa Cruz, USA) (1:50). Cover slips were mounted with ProLong antifade containing DAPI (Vectashield, Biovalley). Specimens were imaged using Leica SP8 laser-scanning microscope equipped with immersion objective 63× plan Apo-NA 1.4 driven by the LAS software. In some experiments permeabilized HeLa cells were labeled with ActinRed™ (Thermo Fisher). Apoptotic cells were detected after DAPI staining with a Zeiss fluorescence microscope using ×400 magnification.

### High content screening analysis

Analysis was performed inside the 96-wells plates^65^. Cells were treated as samples for confocal microscopy using rabbit anti-ɣ-H2AX antibody (1:500, Cell signaling). Immunolabellings were revealed with goat anti-rabbit antibody coupled with Alexa Fluor 555 (1:500, Cell Signaling) in combination with DAPI. DNA and ɣH2AX-immunofluorescence were visualized using a Cellomics ArrayScan VTI HCS Reader (Thermo Fisher) ImPACcell technologic platform. Ten images per well were analyzed and an arbitrary immunofluorescence value was found for each nucleus.

### Flow cytometry analysis

For cell cycle analysis, combined detached and adherent cells were fixed in 70% ethanol overnight, stained with propidium iodide (Sigma-Aldrich) and analyzed with an Accuri C6 flow cytometer. Data were collected from 20, 000 cells, and analysed with CFlow software (Becton Dickinson)^36^.

For ɣH2AX estimation, cells were exposed to *S. aureus* for 2 h and were fixed in 4% paraformaldehyde/PBS followed by the permeabilization in 0.1%Triton/0.5% BSA/PBS. Cells then were incubated with Alexa Fluor 647 mouse anti-ɣH2AX (p139) antibody for 45 min and were analyzed as described above. The percent of relative phosphorylation was calculated as fold changes over the control, which was considered as 100%, and multiplied by 100. In some experiments, cells were treated with 10 mM ROS inhibitor NAC (N-acetyl-L-cysteine) (Sigma-Aldrich) 1 h before the infection. A viability and a metabolic activity of *S. aureus* were not affected by the treatment with NAC since there were no differences between the OD and CFUs of bacterium exposed or not to NAC.

### Preparation of lipoprotein-enriched fraction

Bacteria were cultivated in B-medium (1% soy peptone, 0.5% yeast extract, 0.5% NaCl, 0.1% glucose and 0.1% K_2_HPO_4_, pH 7.4) at 37 °C for 16 h in aerobic conditions. Bacteria were harvested by centrifugation, washed with TBSE buffer (20 mM Tris-HCl, pH8, 130 mM NaCl, 5 mM EDTA) and re-suspended in TSBE buffer with protease inhibitors (Merck) and lysostaphin (30 µg/ml) and incubated at 37 °C for 2 h. Cells then were disrupted with glass beads by using FastPrep-24 instrument. After the centrifugation (4000 × g, for 5 min), the supernatant was mixed with TritonX114/Tris-HCl. The solution was agitated at 4 °C for 1 h and was left to stand for 10 min at 37 °C for phase separation. The lower-phase was collected by centrifugation (10000 × g, for 10 min), mixed with 2.5 fold volume of 100% ethanol and was kept at −20 °C overnight, then the lipoprotein-enriched fraction was precipitated by centrifugation at 13000 × g for 10 min. The pellet was washed with 80% ethanol, dried, dissolved in SDS running buffer, heated for 5 min at 95 °C and loaded on SDS-PAGE.

## Western Blot Analysis

### Bacterial samples

The lipoprotein-enriched fractions were separated by 12% SDS- PAGE and transferred onto nitrocellulose membrane. After overnight incubation with RotiBlock (Roth, Karlsruhe) membranes were incubated with anti-Lpl1-his rabbit antibodies developed by us^66^ followed by incubation with anti-rabbit goat alkaline phosphatase conjugated antibodies (Sigma). Detection was carried out using BCI/NBP (Sigma).

### Eukaryotic cells samples

Immunoblotting of eukaryotic cell lysates was performed as described^67^. HeLa cells were infected with MW2 (MOI 1:50) for 6 and 20 h. Cell pellets were resuspended in buffer containing 150 mM NaCl, 50 mM Tris–HCl, pH 8.0, 0.1% triton, 0.1% SDS and protease inhibitors (Merck). Protein concentration of lysates was determined using BCA protein assay (Interchim). Equal amounts of protein were separated by14% SDS-PAGE followed by the transfer onto 0.2 µM PVDF membranes. The membranes were blocked with 10% skimmed milk then were incubated with rabbit anti-human caspase-3 antibody (Abcam, UK), or with rabbit anti-human PARP (Poly (ADP-ribose) polymerase) antibody (Cell Signaling technology), (1:400) followed by the incubation with peroxidase-labeled goat anti-rabbit antibody (1:1000) (Sigma). Bands were visualized with ECL kit (Pierce, Illkirch, France). The image was processed using a GBOX imaging system (Syngene, Ozyme, Poitiers, France). To assess the quantity of loaded protein, membranes were re-probed with a mouse anti-α-tubulin antibody (Sigma) (1:1000).

### Statistical analysis

Three assays were performed per experiment. The differences among the groups were assessed by ANOVA. P ≤ 0.05 was considered significant. Tukey’s honestly significant difference test was applied for comparison of means between groups. The values are expressed as mean ± SD.

### Ethical approval

*S. aureus* clinical isolates were collected with the approval of the French South-East ethics committee (No. CAL2011-21). In accordance with the French legislation, written informed patient consent was not required, all samples were anonymized.

## Supplementary information

Supplementary information Berkova SR revision

## Data Availability

All data generated or analysed during this study are included in this published article and its Supplementary Information Files.
