# Supplementary material for: Staphylococcus aureus induces DNA damage in host cell
Source: Sci Rep. 2019 May 22;9:7694. doi: 10.1038/s41598-019-44213-3 (PMC6531466; doi:10.1038/s41598-019-44213-3)
Supplement: Supplementary file 1 — Supplementary information Berkova SR revision [file 41598_2019_44213_MOESM1_ESM.pdf]

Staphylococcus aureus induces DNA damage in host cell

Martine Deplanche[1,¶], Nassim Mouhali[1¶], Minh-Thu Nguyen[2], Chantal Cauty[1], Frédéric Ezan[3], Alan Diot[4,5], Lesly Raulin[6], Stephanie Dutertre[6], Sophie Langouet[3], Patrick Legembre[7], Frederic Taieb[8], Michael Otto[9], Frédéric Laurent[4,5], Friedrich Götz[2], Yves Le Loir[1], Nadia Berkova[1*]

[1]STLO, INRA, Agrocampus Ouest, Rennes, France

[2]Microbial Genetics, University of Tübingen, Tübingen, Germany

[3] University of Rennes, Inserm, EHESP, UMR_S 1085 IRSET, Rennes, France

[4]Centre International de Recherche en Infectiologie, INSERM U1111, CNRS, Université Lyon 1, Lyon, France

[5]Centre National de Référence des Staphylocoques, Lyon, France

[6], CNRS, Inserm, BIOSIT-UMS 3480, MRic, Université de Rennes, Rennes, France

[7]Centre Eugène Marquis, Equipe Ligue Contre Le Cancer, Rennes, France.

[8]IRSD, Université de Toulouse, INSERM, INRA, ENVT, UPS, Toulouse, France

[9]Laboratory of Human Bacterial Pathogenesis, US National Institutes of Health, Bethesda, Maryland 20892, USA.

Supplementary information

Supplementary Figure S1
Supplementary Figure S2
Supplementary Table S3
Supplementary Figure S4
Supplementary Figure S5
Supplementary Figure S6
Supplementary Table S7

Supplementary Figure S1. *S. aureus* MW2 strain does not induce apoptosis in HeLa cells in presented experimental conditions

Hela cells were exposed to *S. aureus* MW2 at MOI 1:50 for 2 h or to 1µM of staurosporine as the positive control. Apoptosis was examined 6 h and 20 h post-infection either by Western blot analysis (A, B) or by Fluorescence microscopy (C). Cell extracts were used for the detection of Caspase-3 processing (A) and cleavage of PARP (Poly (ADP-ribose) polymerase) (B) by Western blot analysis. Each membrane, after examination of the caspase-3 processing and PARP cleavage was re-blotted with anti-tubulin antibody. Anti-caspase-3 antibody detected pro-caspase (37 kD) and processed forms of caspases (17 kD) as indicated by arrows. Anti-PARP antibody identified uncleaved 116 kD and cleaved 85 kD form of PARP. A representative experiment from the three is shown.

Nuclear morphology was analyzed by fluorescent microscopy (C). The condensation of chromatin and formation of apoptotic bodies were assessed after staining of the nuclei with DAPI. Samples were viewed with a Zeiss fluorescence microscope using ×400 magnification. Arrows indicate apoptotic bodies

Supplementary Figure S2. Exposure to *S. aureus* induces DNA damage in MG-63 cells.

High Content Screening analysis of *S. aureus* (MW2)-infected vs control non-infected MG-63 cells. Etoposide-treated cells were used as the positive control. Cells were infected for 6 h and 20 h and all cell cultures were treated with gentamicin and lysostaphin as described above. Immunolabelling of phosphorylated γH2AX with the Ɣ-H2AX antibody followed by incubation with the secondary antibody coupled with Alexa Fluor 555 (red staining) in MG-63 cells exposed to *S. aureus* for 6 h and 20 h compared with that of the non-infected control cells and the cells treated with 50 µM etoposide. Nuclei were stained with DAPI (blue staining). Nuclei of etoposide-treated cells demonstrated a high level of DNA containing phosphorylated γH2AX (red staining). Arrows show the site of phosphorylated γH2AX. Fluorescence images were obtained with a Cellomics ArrayScan VTI HCS Reader. Scale bar: 5 µm.

Supplementary Table S3. *S. aureus* induces a dose-dependent DNA damage in MG-63 cells

Relative phosphorylation was obtained using flow cytometry analysis. Normalization was performed as follow: percent of relative phosphorylation of clinical isolates was calculated as fold changes over the one of the control cells that was considered as 100% and multiply by 100.

*P ≤0.05 vs. control cells

Supplementary Figure S4. LpL dampens *S. aureus*–induced host DNA damage

HeLa cells were infected either with USA300 carrying the vSaα island (red), or with the deletion mutant USA300Δlpl (blue) or with the complemented mutant USA300Δlpl (pTX30-*lpl*) (yellow) at MOI 1:25 or 1:50 for 2 h as described. At 20 h post infection phosphorylated γH2AX was quantified by flow cytometry. The data were collected from 20,000 cells and analysis was performed with Cell Quest software. The relative phosphorylation of the control cells (gray) was considered as 100%. Percent of the relative phosphorylation of samples was calculated as fold changes over the control and multiplied by 100. Data are presented as mean ± SD from three independent experiments. According to three independent experiments, exposure of HeLa cells to USA300 (MOI 1:50) resulted in an increase of normalized fluorescence related to ɣH2AX phosphorylation from

100% (control) to 110±4%. Exposure of HeLa cells to the deletion mutant USA300Δ*lpl* (MOI 1:50) increased normalized fluorescence up to 127±5%, while exposure to the complemented mutant USA300Δ*lpl* (pTX30-*lpl*) (MOI 1:50) decreased the normalized fluorescence to 111±4%, at the level of the USA300 wt. The effect was dose-dependent.

Supplementary Figure S5. *S. aureus* recurrent isolates induce stronger DNA damage then initial isolates in MG-63 cells

Osteoblast-like MG-63 cells were exposed for 2 h to tree couples of isolates (MOI 1:50) that have been recovered from three patients (P1, P2, P3) at the time of the initial (45i, 47i, 51i) and relapsing (46r, 48r, 52r) infection from P1, P2, P3 correspondently. At 20 h post-infection phosphorylated γH2AX was monitored by High Content Screening approach that combines automated imaging and quantitative data analysis as described in Material and Methods. DNA and ɣH2AX-immunofluorescence were visualized using a Cellomics ArrayScan VTI HCS Reader ImPACcell technologic platform. The images from one representative experiment are demonstrated.

Supplementary Figure S6. *S. aureus* recurrent isolates express a lower amount of Lpls than initial acute isolates

S. aureus strains isolated from three patients (P1, P2, P3) with initial (45i, 47i, 51i) and recurrent (46r, 48r, 52r) BJI from P1, P2, P3 correspondently were maintained as described. Lpls enriched fractions were prepared as indicated in Material and Methods. TritonX114 lipoprotein-enriched fractions, prepared from 6 clinical isolates, were separated on 12% SDS-PAGE: (**a**) Gel was stained with Coomassie blue (**b**) The Western blot analysis was performed using anti-Lpl1-his antibody which we developed. Molecular Weight markers are presented at the left side of SDS-PAGE gel and membrane. The arrow indicates *S. aureus* LpLs.

Supplementary Table S7. *S. aureus* strains from patients with a relapsing BJI induce a stronger G2/M phase transition delay than isolates from patients with an initial BJI

Distribution of cell cycle phases of HeLa cells synchronized by DTB was obtained using flow cytometry analysis.

$^{\Delta}$ Cells complete the first cell cycle and progress within the second cell cycle.

*P ≤ 0.05 cells exposed to initial vs. cells exposed to recurrent clinical isolate from the same patient.

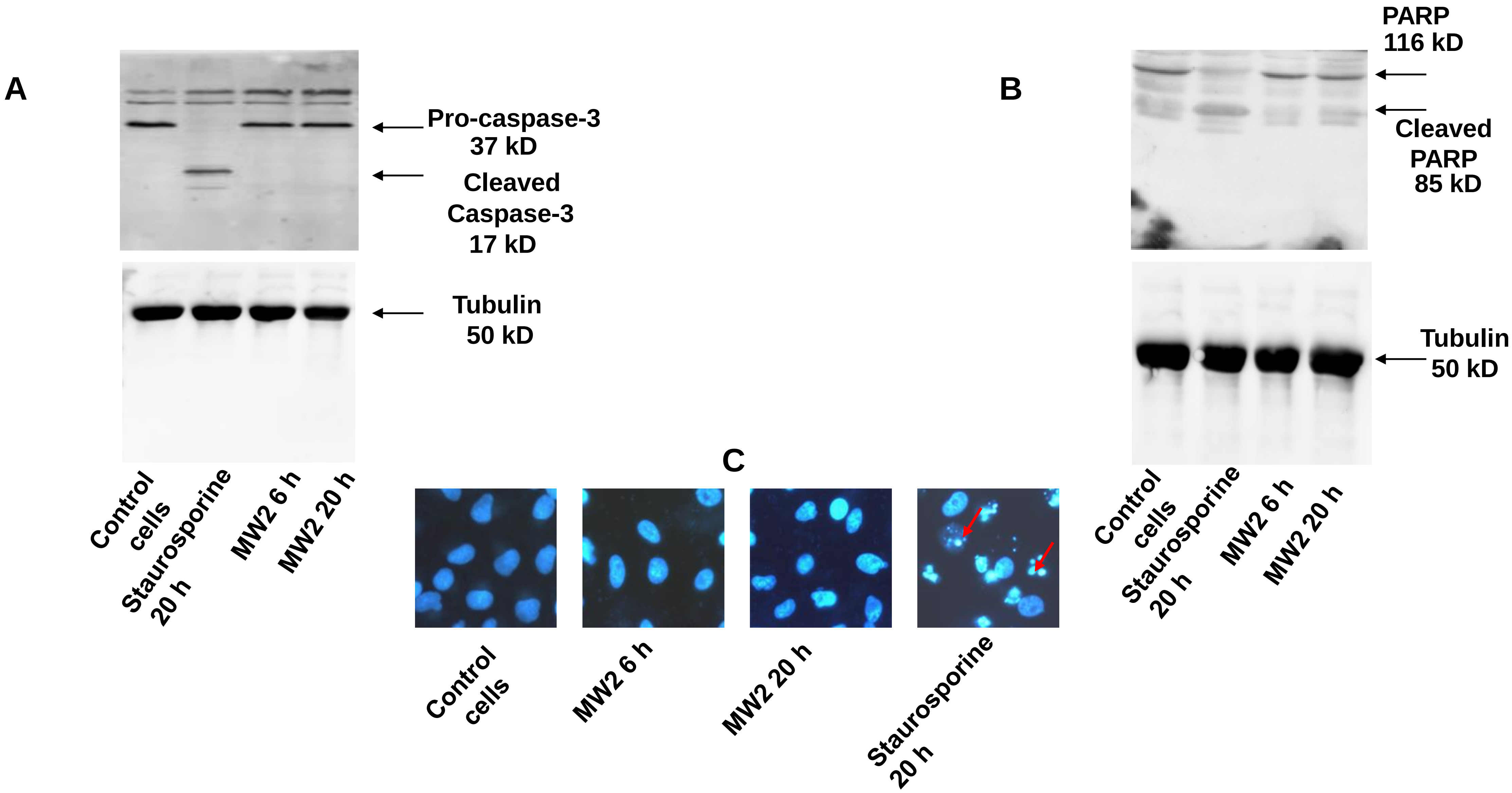

Supplementary Figure S1

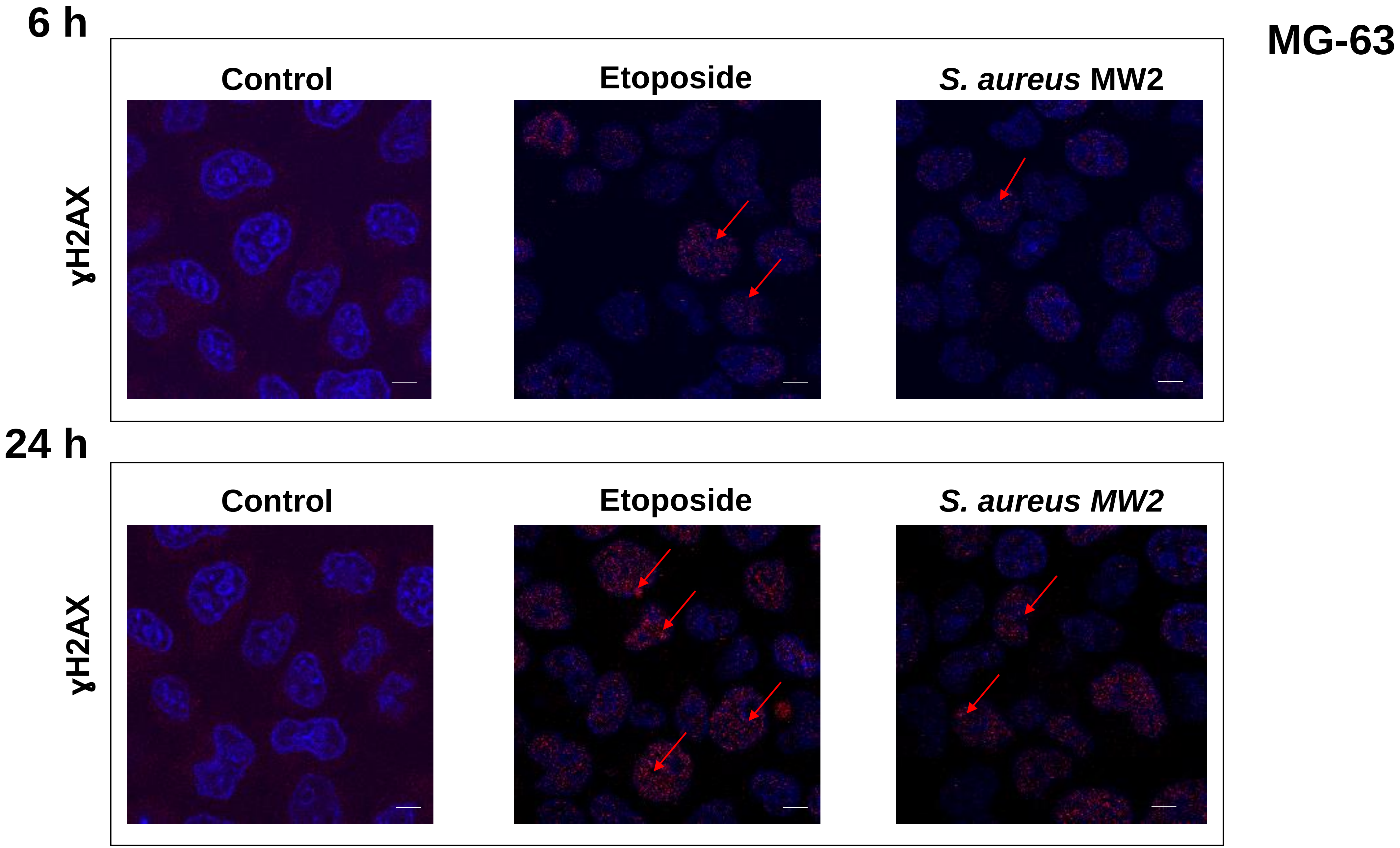

Supplementary Figure S2

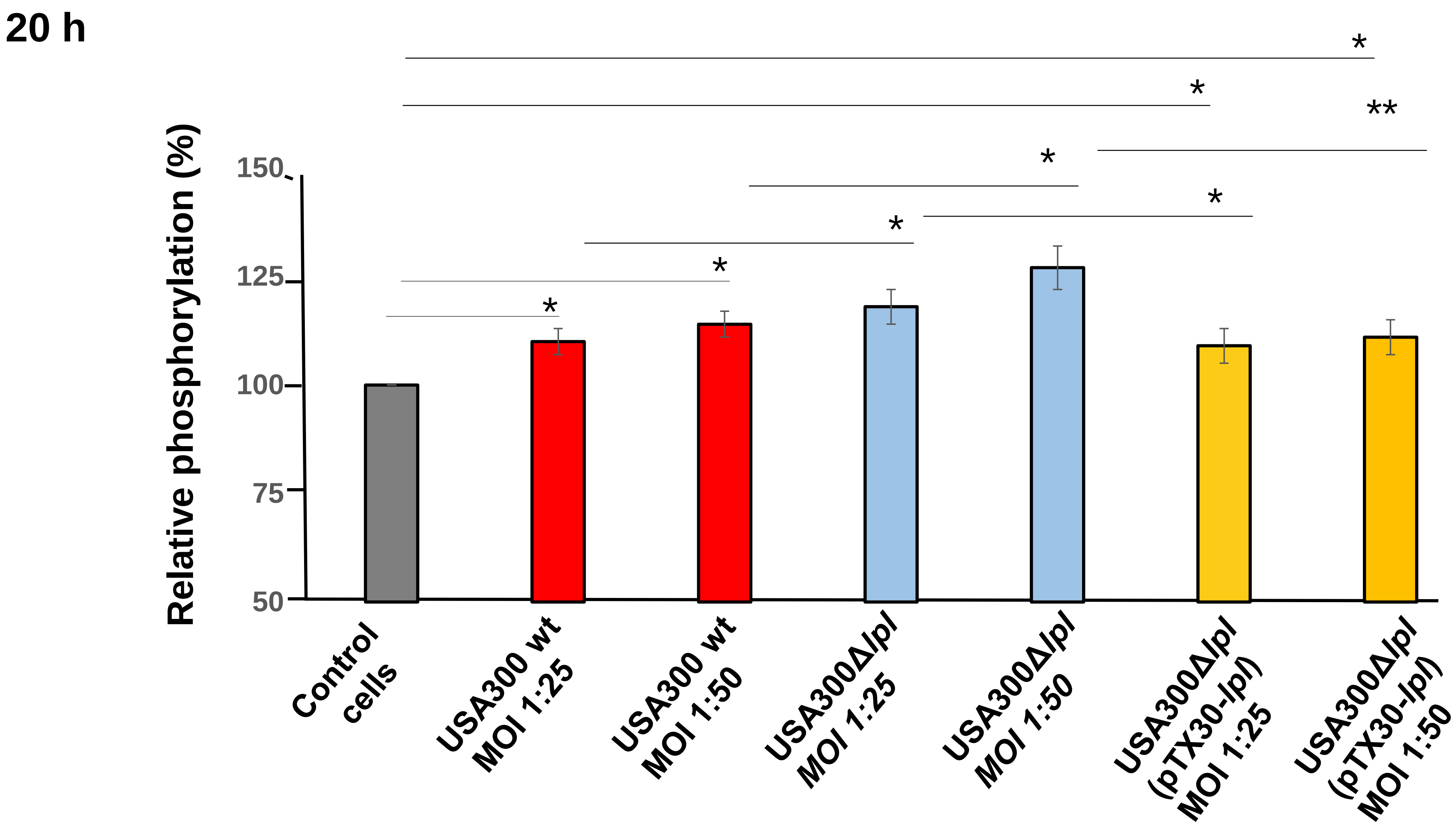

Supplementary Figure S4

MG-63

20 h

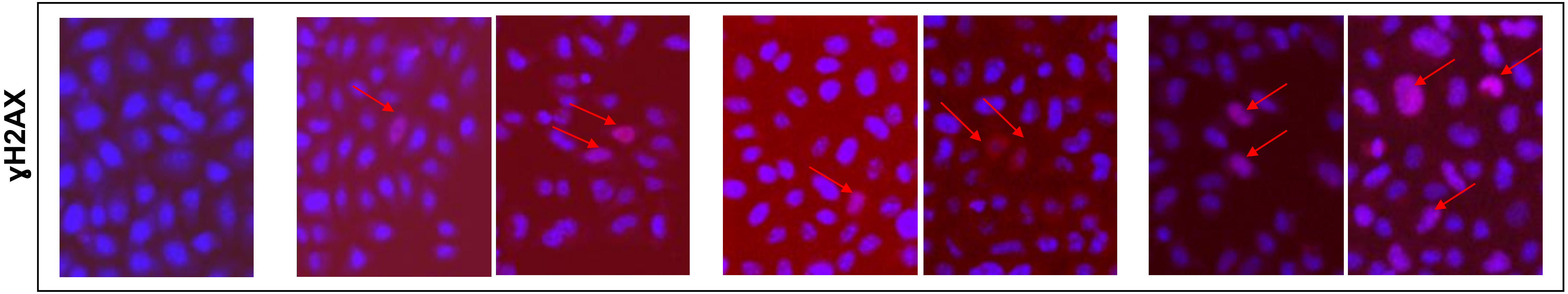

Control

Patient 1

45i 46r

Patient 2

47i 48r

Patient 3

51i 52r

Supplementary Figure 5

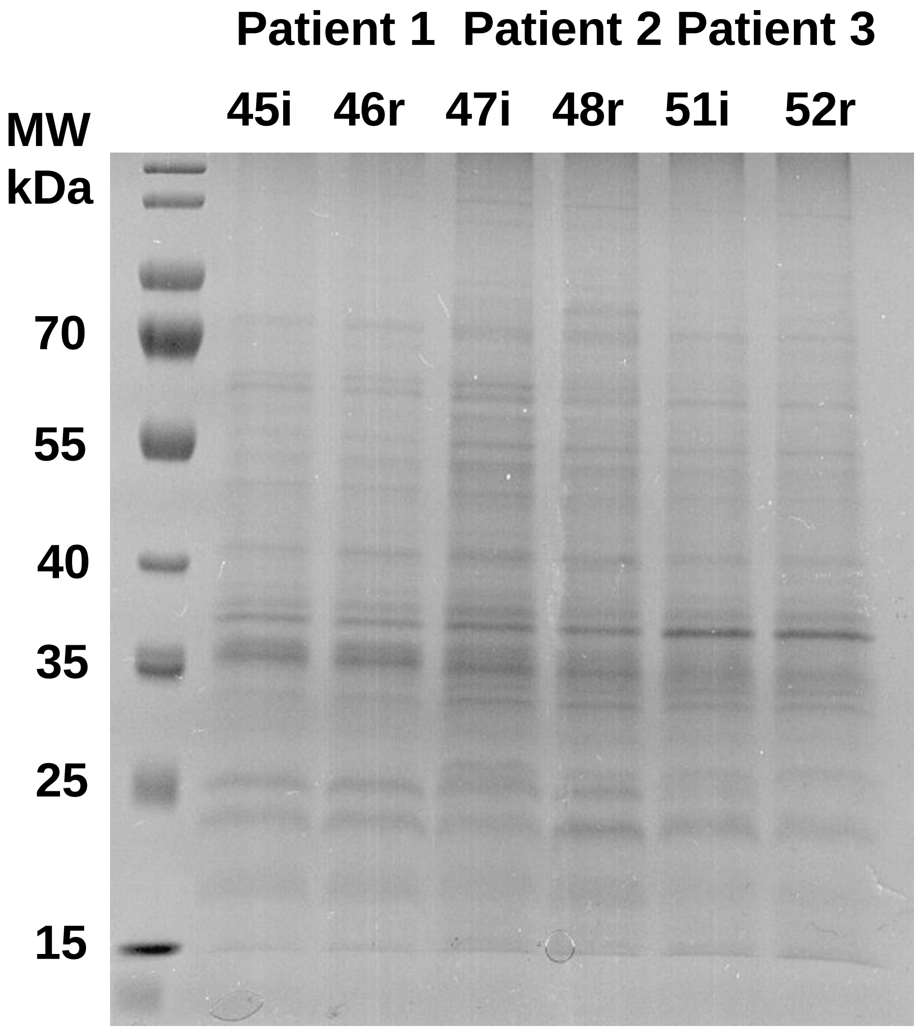

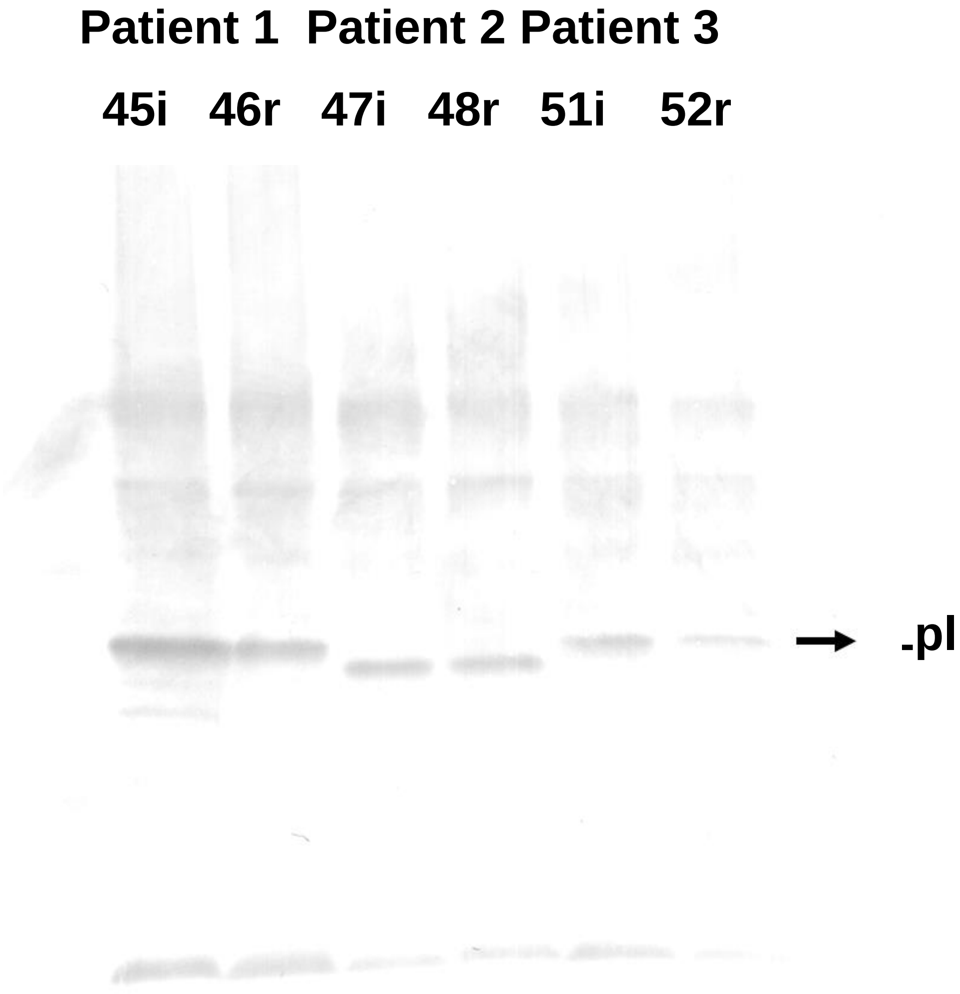

Supplementary Figure S6

Supplementary Table S3

S. aureus **induces a dose-dependent DNA damage in MG-63 cells**

Conditions / Samples	Relative phosphorylation 6 h		Relative phosphorylation 20 h	
	MOI 1 :25	MOI 1 :50	MOI 1 :25	MOI 1 :50
Non-infected MG-63 cells	100%		100%	
MG-63 cells + *S. aureus* (MW2)	108±4%	118±6%*	113±5%	125±6%*

Supplementary Table S7
***S. aureus* strains from patients with a relapsing BJI induce a stronger G2/M phase transition delay than isolates from patients with an initial BJI**

Samples	G1%	S%	G2/M%
**Control$^{\Delta}$**	38±6.1$^{\Delta}$	34±3.9$^{\Delta}$	28±4.3$^{\Delta}$
HeLa cells+ strain 45i	23±5.1	27±4.3	50±4.4*
HeLa cells + strain 46r	19±5.8	17±4.5	64±6.1*
HeLa cells + strain 47i	48±5.5	14±4.9	38±6.1*
HeLa cells + strain 48r	40±5.4	13±4.1	47±5.7*
HeLa cells + strain 51i	27±5.6	32±5.0	41±4.2*
HeLa cells strain 52r	16±5.4	16±4.1	68±5.7*
